# Improved wood species identification based on multi-view imagery of the three anatomical planes

**DOI:** 10.1186/s13007-022-00910-1

**Published:** 2022-06-11

**Authors:** Núbia Rosa da Silva, Victor Deklerck, Jan M. Baetens, Jan Van den Bulcke, Maaike De Ridder, Mélissa Rousseau, Odemir Martinez Bruno, Hans Beeckman, Joris Van Acker, Bernard De Baets, Jan Verwaeren

**Affiliations:** 1 KERMIT, Department of Data Analysis and Mathematical Modelling, Ghent University, Ghent, Belgium; 2 Institute of Mathematics and Computer Science, University of São Paulo, São Carlos, Brazil; 3 Present Address: Institute of Biotechnology, Federal University of Catalão, Catalão, Goiás Brazil; 4 Royal Botanic Gardens Kew, Richmond, Surrey, UK; 5 Laboratory of Wood Technology, Department of Environment, Ghent University, Ghent, Belgium; 6 Service of Wood Biology, Royal Museum for Central Africa, Tervuren, Belgium; 7 São Carlos Institute of Physics, University of São Paulo, São Carlos, Brazil

**Keywords:** Wood species identification, Wood anatomical sections, Texture analysis, Machine vision, Machine learning

## Abstract

**Background:**

The identification of tropical African wood species based on microscopic imagery is a challenging problem due to the heterogeneous nature of the composition of wood combined with the vast number of candidate species. Image classification methods that rely on machine learning can facilitate this identification, provided that sufficient training material is available. Despite the fact that the three main anatomical sections contain information that is relevant for species identification, current methods only rely on transverse sections. Additionally, commonly used procedures for evaluating the performance of these methods neglect the fact that multiple images often originate from the same tree, leading to an overly optimistic estimate of the performance.

**Results:**

We introduce a new image dataset containing microscopic images of the three main anatomical sections of 77 Congolese wood species. A dedicated multi-view image classification method is developed and obtains an accuracy (computed using the naive but common approach) of 95%, outperforming the single-view methods by a large margin. An in-depth analysis shows that naive accuracy estimates can lead to a dramatic over-prediction, of up to 60%, of the accuracy.

**Conclusions:**

Additional images from non-transverse sections can boost the performance of machine-learning-based wood species identification methods. Additionally, care should be taken when evaluating the performance of machine-learning-based wood species identification methods to avoid an overestimation of the performance.

**Supplementary Information:**

The online version contains supplementary material available at 10.1186/s13007-022-00910-1.

## Background

### Illegal wood trade and wood species identification

Illegal logging is the most profitable natural resource crime and illegal wood accounts for 10 to 30 percent of the total global trade in wood products [1, 2], and increasing up to 50 and 90 percent when focusing on Southeast Asia, Central Africa, and South America [1]. The financial value of illegal logging is estimated at US$52 to 157 billion dollars per year. There is also a high risk of irreversible damage to ecosystems associated with the exploitation of highly sought after, sometimes protected, species. To prevent the over-exploitation of these species, protection measures are put in place, for example the Convention on International Trade in Endangered Species of Wild Fauna and Flora [3]. In addition, policy measures [for example, EUTR (European Union Timber Regulation) 2013 and U.S. Lacey Act] are implemented in countries to counter the trade in illegal wood and to improve forest law enforcement and governance [4].

To enforce these regulations and policy measures, wood species identification techniques combined with robust datasets are needed. Wood species identification is currently mainly done via wood anatomical analysis and DART-TOFMS (Direct Analysis in Real Time (DART™) ionization coupled with Time-of-Flight Mass Spectrometry), proven to be successful in routine controls, and there are other viable techniques as well, for example DNA analysis and Near InfraRed spectroscopy [5–13]. Wood anatomical analysis is the most widely applied, readily available and least expensive technique. Identification is possible via an analysis of tissue and cell features through hand lenses, light or electronic microscopes or 2D or 3D scans and the IAWA list of microscopic features [14]. The IAWA characteristics are based on patterns of anatomical features, such as vessels, rays, parenchyma and fibres. This approach is usually sufficient to identify the genus, but sometimes fails to determine the species [15, 16]. Moreover, it can be difficult to discern between closely related taxa.

### Automated identification through wood anatomical images

Wood anatomical analysis is a complicated task that can take several years to master and will always involve expert knowledge. Driven by the success of automation of image recognition in other fields, several attempts have been made to automate wood species identification using computer vision models that use digital imagery of anatomical sections as input. The construction of these models is mostly handled as a pattern recognition task in which: (1) a representative dataset of labeled digital images is collected (the label is the species); (2) a feature extraction procedure is applied; and (3) a machine learning classification algorithm is trained to discriminate the species using the features. The approaches found in literature mostly differ by the choices that are made within each of these steps. We present an overview hereafter.

Martins et al. [17] used an image dataset consisting of 112 species, a large number compared to other studies, including both hardwood and softwood species with a total of 2240 or approximately 20 images of microscopic transverse images per species. The authors experimented with different feature descriptors and concluded that Local Binary Patterns (LBP) as a feature (texture) descriptor combined with Support Vector Machines (SVMs) as a classification algorithm yields the best performance. They reported an accuracy of 86.0%. Filho et al. [18] composed an image dataset containing 41 Brazilian species with a total of 2942 macroscopic transverse images. They adopted a strategy where first the image is divided into sub-images which are then classified independently. A different feature extractor is applied to each sub-image, resulting in separate feature vectors. Subsequently, a SVM (a probabilistic variant is used) is trained on each feature vector. The class probabilities that are predicted by the individual SVMs are aggregated through a fusion rule to obtain a final prediction. For the 41 species they reached an accuracy of 97.77%. Rosa da Silva et al. [19] used a dataset containing 1221 microscopic images of 77 commercial wood species from the Democratic Republic of the Congo. They used Local Phase Quantization (LPQ) as a feature descriptor and linear discriminant analysis as a classifier, resulting in an accuracy of approximately 88% at species level. Ravindran et al. [20] composed a dataset containing 2303 macroscopic images of 10 species from the Meliaceae family. They used (deep) convolutional neural networks (CNN) as a classifier. The convolutional layers serve as data-driven feature extractors, obviating the need for feature descriptors. They obtained an accuracy of 87.4%. Recently, Souza et al. [21] used LBP in the construction of an automated recognition system of Brazilian forest species. Forty six species were used in their analysis, with a total of 1901 macroscopic images. An automatic recognition system based on the concatenation of rotation-invariant LBP histograms and an SVM classifier obtained an F1-score of 97.67%. This approach requires a large reference dataset that captures all potential variability within a species [15]. However, thanks to historical wood collections (see also the Index Xylariorum 4.1 [22]), there are many curated wood anatomical slices available that can be used as a reference for identification. There is also the online wood anatomical imagery dataset InsideWood [23], which is the most extensive dataset of species descriptions and microscopic images based on IAWA characteristics.

Similarly, Ravindran et al. [24] used CNNs to identify 12 self-defined classes based on macroscopic imagery of transverse sections of species that are common in the United States. Using a training dataset containing 3126 images, they obtained an accuracy of 97.7%. Along that line, Lens et al. [25] reported a similar accuracy (over 98% using CNN) on 2240 microscopic images of transverse sections of 112 species.

The literature reviewed above illustrates that machine-vision-based wood species identification systems can, in some cases, reliably identify wood species. However, there is still room for improvement at several levels. First, the machine vision systems described in literature only use images of the transverse anatomical plane. The tangential and radial anatomical planes can also include information that is relevant for the species identification. For example, the height, width and organization (storied or not) of the rays can be important characteristics that can only be seen on tangential and radial planes (see also Gasson et al. [8]). To this date, there are no generally available image datasets that contain imagery of the different anatomical planes. Although InsideWood [23] offers a big image database, these images cannot be readily downloaded as a batch and have different magnifications. To fill this gap, we introduce a new multi-view dataset. Secondly, we propose to use the taxonomy of the considered species to build a hierarchical classifier. For classifiers that output a probability distribution over the species, the Bayesian optimal decision criterion based on a hierarchical cost function can be used to encode this hierarchy into the identification problem. Third, in most research, cross-validation approaches are used to assess the performance of the developed systems. However, it is not always clear how cross-validation procedures are applied. Most publications mention that traditional *k*-fold (possibly stratified at the species level) is used. It is important to note that imagery datasets often result from a limited number of distinct trees. When these images are used in a traditional (random) *k*-fold cross-validation scheme, the performance can be overestimated.

This potential shortcoming is also explicitly mentioned in [25] as a source of potential underestimation of intra-species variability, where the authors state that they were unable to trace back images to individual samples using the dataset of [17]. In this work, we critically compare the performances obtained using a traditional *k*-fold approach with those obtained using a leave-*k*-tree-out approach. Therefore, the purpose of this paper is threefold. (1) We introduce a new image dataset that contains images of the three anatomical planes of 77 Congolese wood species and propose a multi-view random forest model that can identify a specimen at the species level using images of the three anatomical planes. We compare the performance of this multi-view approach with the performance that is obtained when using only the transverse section. (2) We incorporate information on the higher taxonomic level (genus and family) into the classification model by post-processing the probability estimates of random forest models. (3) We study the influence of using a leave-*k*-tree-out approach during cross-validation.

## Method

### Compilation of a multi-view image dataset

Datasets that contain imagery of the three anatomical planes of wood samples are not readily available for the purpose of the type of analysis we intend to perform in this paper, with standardized preparation of all samples. We introduce a new image dataset containing images of the three anatomical planes of 77 Congolese species. Note that this dataset is an extension of the dataset used in [19]. The wood samples were collected in the Democratic Republic of the Congo and the wood anatomical slices were prepared by the Service of Wood Biology at the Royal Museum for Central Africa (Tervuren, Belgium). The sections were cut with a sliding microtome, dehydrated in a graded ethanol series (50%, 75%, 96% and 100%) and fixed with Euparal. A light microscope (Olympus BX60) in connection with a digital camera (Olympus UC30) and the image analysis software package CellB (version 3.2, Olympus) were used to acquire RGB images with 2.5× standard magnification. The images were cropped to size 1000 × 1000 pixels for processing corresponding to 1388.88 × 1388.88 $\upmu$m.

**Fig. 1:**
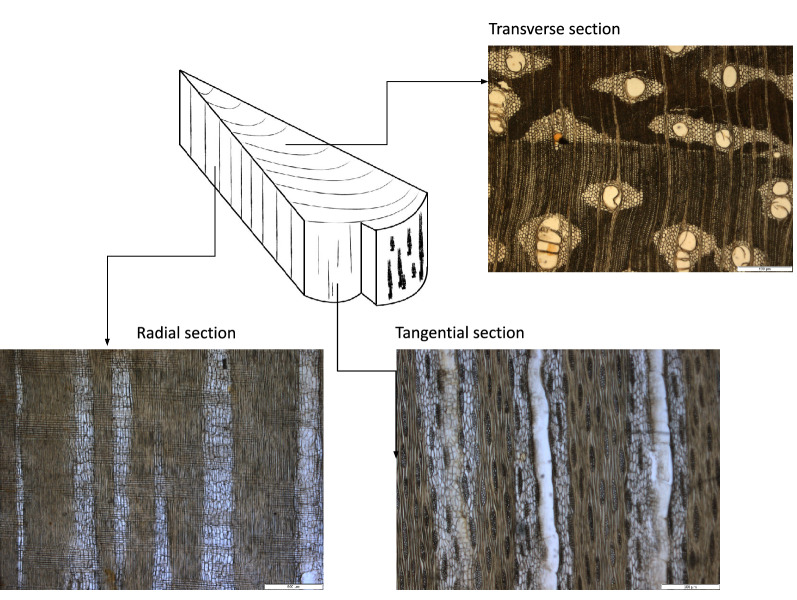
Image acquisition of wood transverse, tangential and radial sections. Text of the scale bar: 500 $\upmu$m

**Fig. 2:**
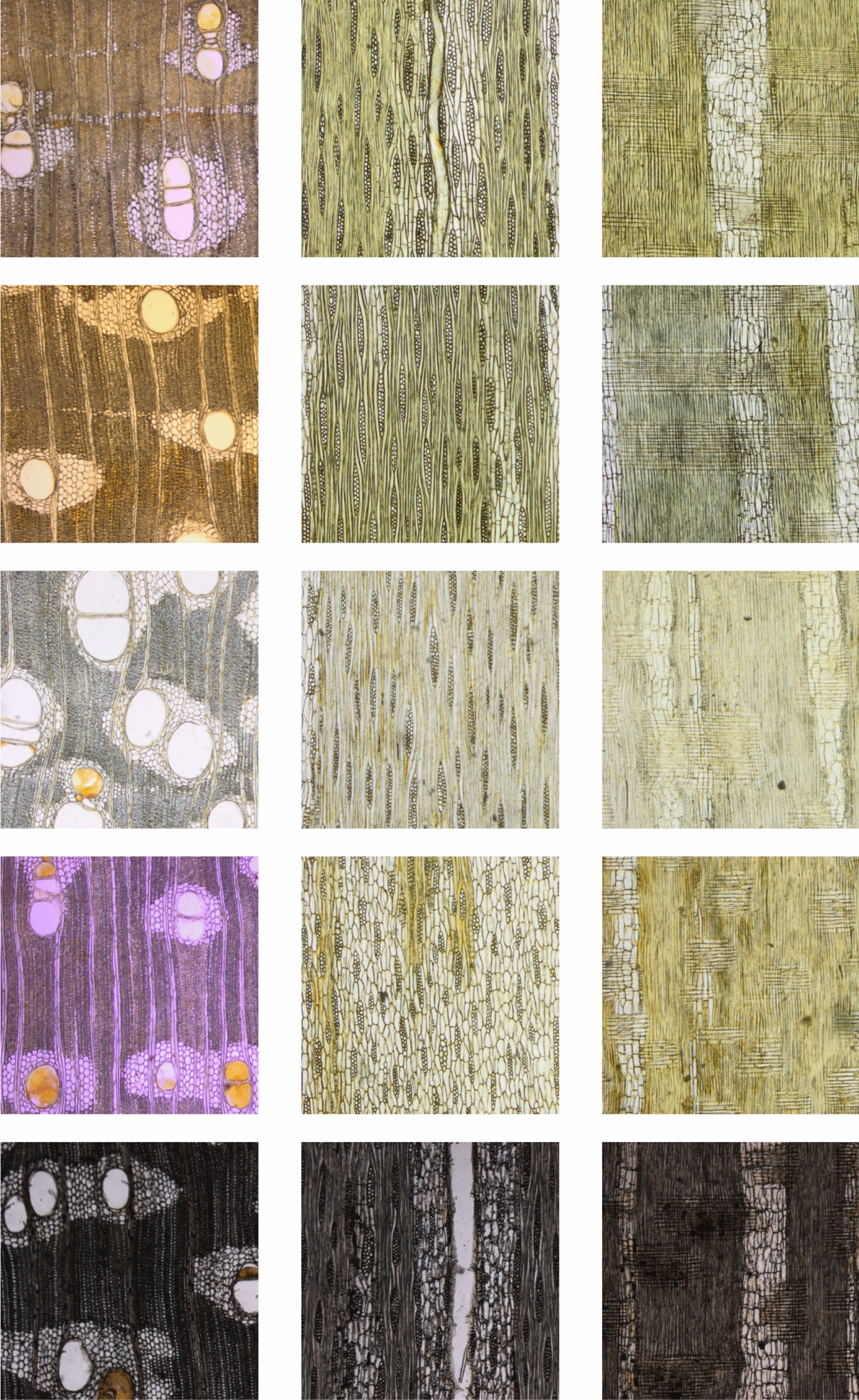
Samples of the wood image dataset showing in each column: transverse, tangential and radial sections. Each row shows a single species with the three planes, being, from top to bottom: *Afzelia africana*, *Afzelia bella*, *Afzelia bipindensis*, *Afzelia quanzensis* and *Afzelia pachyloba*. Each image has 1000 × 1000 pixels corresponding to 1388.88 × 1388.88  $\upmu$m

One wood slice generates three images, i.e., one image for each distinct cross sectional surface of the tree trunk: transverse, tangential and radial, as shown in Fig. 1. The transverse anatomical section runs at right angles to the main axis of the stem or the trunk. The tangential section cuts across the rays of a block of wood or a stem, while the radial section runs parallel to the rays. All together, 805 × 3 = 2415 images belonging to 77 species, 58 genera and 25 families were obtained (see Table 1). Figure 2 shows samples from five species of the genus Afzelia.

**Table 1:** Species and families included in the analysis

Species	Family	Samples
*Afzelia africana*	Fabaceae–Detarioideae	9
*Afzelia bella*	Fabaceae–Detarioideae	5
*Afzelia bipindensis*	Fabaceae–Detarioideae	8
*Afzelia quanzensis*	Fabaceae–Detarioideae	8
*Afzelia pachyloba*	Fabaceae–Detarioideae	8
*Albizia adianthifolia*	Fabaceae–Caesalpinioideae–Mimosoid-clade	17
*Albizia antunesiana*	Fabaceae–Caesalpinioideae–Mimosoid-clade	10
*Albizia ferruginea*	Fabaceae–Caesalpinioideae–Mimosoid-clade	14
*Alstonia boonei*	Apocynaceae	12
*Amphimas ferrugineus*	Fabaceae–Papilionoideae	8
*Amphimas pterocarpoides*	Fabaceae–Papilionoideae	9
*Anthonotha macrophylla*	Fabaceae–Detarioideae	7
*Antiaris toxicaria*	Moraceae–Caesalpiniaceae	12
*Antrocaryon nannanii*	Anacardiaceae	17
*Autranella congolensis*	Sapotaceae	8
*Beilschmiedia congolana*	Lauraceae	10
*Brachystegia laurentii*	Fabaceae–Detarioideae	7
*Canarium schweinfurthii*	Burseraceae	13
*Ceiba pentandra*	Malvaceae–Bombacoideae	6
*Celtis gomphophylla*	Cannabaceae	11
*Chrysophyllum africanum*	Sapotaceae	4
*Chrysophyllum lacourtianum*	Sapotaceae	8
*Copaifera mildbraedii*	Fabaceae–Detarioideae	13
*Cordia platythyrsa*	Boraginaceae	8
*Cynometra alexandri*	Fabaceae–Detarioideae	15
*Cynometra hankei*	Fabaceae–Detarioideae	10
*Diospyros crassiflora*	Ebenaceae	10
*Drypetes gossweileri*	Euphorbiaceae	10
*Ekebergia capensis*	Meliaceae	8
*Entandrophragma angolense*	Meliaceae	20
*Entandrophragma candollei*	Meliaceae	13
*Entandrophragma cylindricum*	Meliaceae	14
*Entandrophragma utile*	Meliaceae	17
*Erythrophleum suaveolens*	Fabaceae–Caesalpinioideae	6
*Ficus mucuso*	Moraceae	8
*Funtumia africana*	Apocynaceae	15
*Gilbertiodendron dewevrei*	Fabaceae–Detarioideae	11
*Guibourtia arnoldiana*	Fabaceae–Detarioideae	8
*Guibourtia demeusei*	Fabaceae–Detarioideae	9
*Mitragyna stipulosa*	Rubiaceae	17
*Holoptelea grandis*	Ulmaceae	12
*Irvingia grandifolia*	Irvingiaceae	14
*Khaya anthotheca*	Meliaceae	14
*Klainedoxa gabonensis*	Irvingiaceae	9
*Leplaea cedrata*^a^	Meliaceae	15
*Leplaea laurentii*^a^	Meliaceae	20
*Leplaea thompsonii*^a^	Meliaceae	5
*Lophira alata*	Ochnaceae	4
*Lovoa trichilioides*	Meliaceae	11
*Mammea africana*	Clusiaceae	10
*Milicia excelsa*	Moraceae	12
*Millettia laurentii*	Fabaceae–Papilionoideae	10
*Morus mesozygia*	Moraceae	7
*Musanga cecropioides*	Moraceae	12
*Nauclea diderrichii*	Rubiaceae	12
*Nesogordonia kabingaensis*	Malvaceae–Dombeyoideae	8
*Newtonia leucocarpa*	Fabaceae–Caesalpinioideae–Mimosoid-clade	7
*Ongokea gore*	Olacaceae	10
*Pentaclethra eetveldeana*	Fabaceae–Caesalpinioideae–Mimosoid-clade	7
*Pentaclethra macrophylla*	Fabaceae–Caesalpinioideae–Mimosoid-clade	9
*Pericopsis elata*	Fabaceae–Papilionoideae	5
*Petersianthus macrocarpus*	Lecythidaceae	11
*Piptadeniastrum africanum*	Fabaceae–Caesalpinioideae–Mimosoid-clade	12
*Pouteria aningeri*	Sapotaceae	8
*Prioria balsamifera*	Fabaceae–Detarioideae	12
*Prioria oxyphylla*	Fabaceae–Detarioideae	14
*Pterocarpus soyauxii*	Fabaceae–Papilionoideae	17
*Pterocarpus tinctorius*	Fabaceae–Papilionoideae	10
*Pycnanthus angolensis*	Myristicaceae	4
*Scorodophloeus zenkeri*	Fabaceae–Detarioideae	8
*Staudtia kamerunensis*	Myristicaceae	13
*Terminalia superba*	Combretaceae	9
*Tessmannia africana*	Fabaceae–Detarioideae	13
*Tieghemella heckelii*	Sapotaceae	9
*Triplochiton scleroxylon*	Malvaceae–Helicteroideae	10
*Zanthoxylum gilletii*	Rutaceae	7
*Zanthoxylum lemairei*	Rutaceae	12

The samples and slices were collected from the Tervuren Wood Collection in the Royal Museum for Central Africa (Belgium)

^a^This species used to be part of the genus Guarea

### Data augmentation

**Fig. 3:**
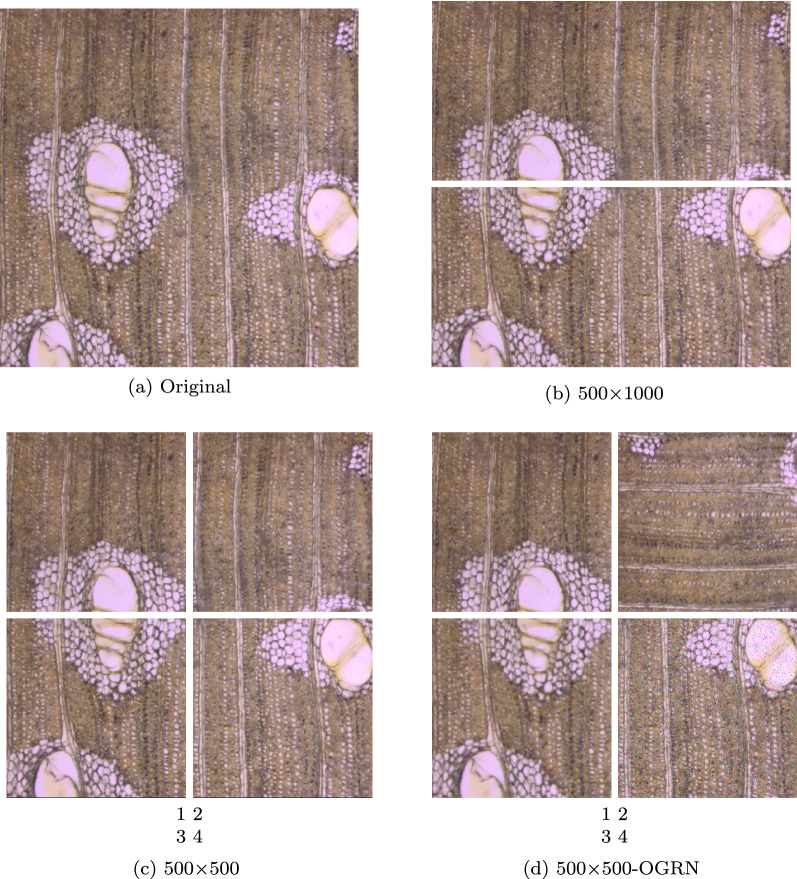
Data augmentation procedure. Images from a sample of *Afzelia africana*. **a** Original image. Original image divided in two parts (**b**) and four parts (**c**). **d** Original image divided in four parts applying a 2-D Gaussian smoothing kernel with standard deviation of 1 at the second piece, rotating the third piece 90 degrees and adding salt-and-pepper noise with a density of 0.05 to the fourth piece. The original image **a** has 1000 × 1000 pixels corresponding to 1388.88 × 1388.88 $\upmu$m

On average only 10 images were available (for each species), which is too few for machine vision applications. Therefore, data augmentation was used to increase the number of samples per species. A first data augmentation step consisted of partitioning the original images (original size 1000 × 1000 pixels, see Fig. 3a). Two options were explored: (1) dividing the original images in half (Fig. 3b), and (2) dividing the images into four parts, resulting in images of 500 × 500 pixels (Fig. 3c). In a second step, augmentation was performed by filtering using a 2-D Gaussian smoothing kernel with a standard deviation of 1, the creation of rotated versions by rotating the original images 90° and the addition of salt-and-pepper noise with a density of 0.05 (Fig. 3d).

All of these actions were performed on the three planes of section. To be able to investigate the influence of this data augmentation step, we kept track of four datasets with images of different sizes: 1000 × 1000 pixels (original), 500 × 1000 pixels (partitioned by dividing in half), 500 × 500 pixels (partitioned by dividing into four parts) and 500 × 500−OGRN (partitioned into four parts, being the first piece, original—O, the second, smoothed—G, the third, rotated—R and the last one, noisy—N). The effect of the data augmentation step on the feature representation of the images (for the species *Afzelia africana*) is shown in Additional file 1: Fig. S1.

### Image preprocessing and feature extraction

To prepare the image data for further analysis, the color images were transformed into grayscale images and digitally enhanced using histogram stretching (1% saturation tolerance). Subsequently, features were extracted from the preprocessed images. In this paper, Local Phase Quantization (LPQ) [26, 27] is used as texture feature descriptor, as in most studies involving wood species identification [17–19, 21]. In total, 256 LPQ features were used.

### Image classification for species identification

#### Single-view classification

Most work on the development of machine learning models for the classification of wood samples based on microscopic imagery relies on a single transverse image of the sample. For that reason, we use this approach as a baseline. More precisely, the random forest algorithm [28] was used to construct a classifier that takes the LPQ features of a transverse image as input and makes a prediction at species level. The *forest* it builds, is an ensemble of decision trees, in our case 500 trees. The number of features (randomly) selected at each split was set to 15. Two additional random forest classifiers were constructed, a first classifier that takes the LPQ features of the radial image as input and a second classifier that takes the features of the tangential image as input. All classifiers were trained independently and evaluated using a cross-validation scheme (see “Results” section).

#### A multi-view random forest model (MVRF)

**Fig. 4:**
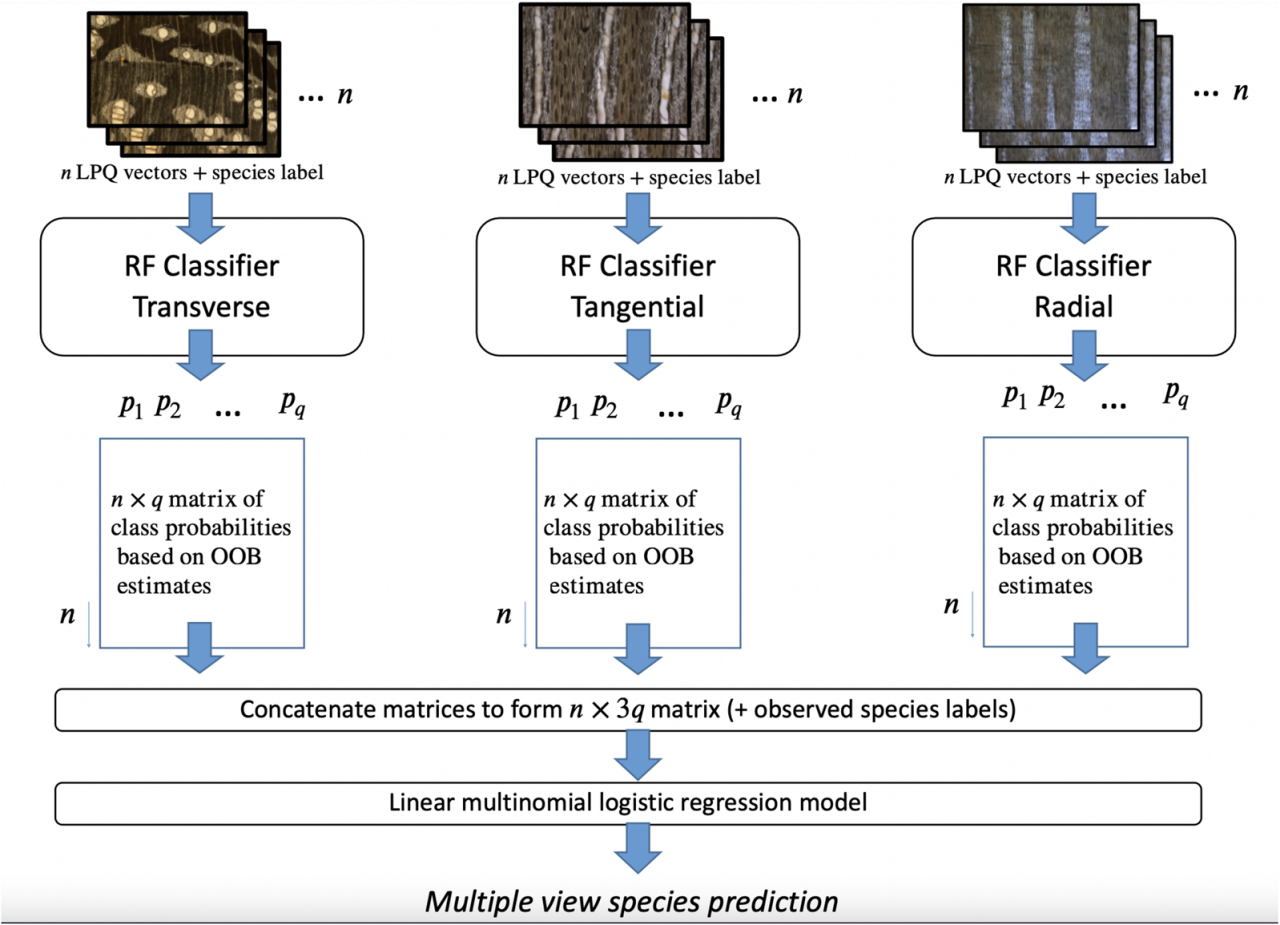
Visualization of the architecture of the multi-view random forest classifier, where *n* represents the number of training observations

The images of the transverse, tangential and radial sections of a wood sample can be interpreted as multiple views of an object. Several options exist that allow to incorporate multi-view imagery in a machine learning model. A first (simple) approach that we explore consists of concatenating the LPQ feature vectors of the three images. In this case the new feature space is the Cartesian product of the three original feature spaces. This approach has at least three potential downsides: (1) the size of the feature space is tripled in a setting that is already data-scarce; (2) the concatenation is agnostic to the fact that the features originate from different images and (3) the concatenation is agnostic to the classification problem at hand. To overcome these problems, we propose a model architecture that extends the basic random forest model and allows for the combination of multiple views and is inspired by the stacking of classifiers (see Fig. 4 for a visualization of the architecture). In a first step a separate random forest model is trained for each of the three views using a training dataset. For each image in the training dataset, the 500 trees in each random forest then each cast a vote for one of the $q = 77$ classes (the species). Per image, the relative frequencies of these votes are subsequently combined into a vector (which is a proxy for the predicted class probabilities). The vectors of the three views that are obtained in this way are then concatenated to form a meta-feature vector. These meta-feature vectors form the inputs of a meta-training dataset (the outputs are the species labels). Subsequently, a multinomial logistic regression model is trained on this meta-dataset to predict the final species label. We conclude this paragraph with a subtle, but important, implementation detail. To obtain the meta-training dataset during training, only out-of-bag votes are used to compute the meta-vector of relative frequencies. Recall that due to the use of bootstrapping, each training observation is used (on average) in only two out of three trees in a forest. As only these trees are allowed to cast a vote, the meta-feature vector will not be prone to overfitting. For allowing this stacking approach to work in practice, the meta-feature vector must be representative for the meta-feature vector of the test instances [29].

#### Leave-*k*-trees-out cross-validation

Traditionally, the performance of a classifier is assessed using a separate training set or *k*-fold cross-validation. The split between test and training set (or the definition of the folds in case of *k*-fold cross-validation) is made using a (stratified) random sampling scheme, with the aim of constructing a test set that is independent from the training set. However, when working with microscopic imagery of wood samples, and especially those originating from historical collections, a single block of wood is often used to make several prepared microscopic slides. As a result, the images originate from the same piece of wood and might show less biological variability as compared to images from different pieces of wood. Moreover, they are often made in sequence and therefore under more similar conditions as compared to slides that are prepared during a period spread out in time, possibly by several lab technicians, and so on. As a result, the image-to-image variability within one piece of wood can be assumed to be smaller than the inter-tree variability. As such, when using a stratified cross-validation scheme with stratification at the species level (or stratified train-test split), images of the same piece of wood can end up in both training and test sets. In this way, these sets cannot be considered independent and performance estimates can be too optimistic. As an alternative, we propose a cross-validation scheme which we call ‘leave-*k*-trees-out scheme’, in which all images that originate from the same tree are either in the training or the test set. In our results section, we compare a traditional cross-validation scheme (in particular the out-of-bag performance estimator of the random forest classifier, which is almost identical to leave-one-image-out cross-validation [30, 31]) and the leave-*k*-trees-out scheme.

#### Including genus and family information in the classification process

In the methods described previously, the accuracy on a test set is used to evaluate the performance of a model. By definition, each misclassified instance has the same influence on the final accuracy. In our (multi-class) species identification problem, it can be argued that this is too simplistic. For example, consider a test instance with true label *y* and predicted label $y^{\prime}$. The case where $y \ne y^{\prime}$ but both labels belong to the same genus may be not such an issue for some applications than the case where *y* and $y^{\prime}$ belong to different genera. Additionally, the cost associated with a misclassification may increase further when *y* and $y^{\prime}$ belong to different families. To generalize this example, we define cost functions for which the cost is determined by the genus or family distance between *y* and $y^{\prime}$. We formally define this cost function as follows:

1 $$C(y, y^{\prime}) = \left\{ \begin{array}{ll} 0, & \text{ if } y = y^{\prime}, \\ 1, & \text{ if } y \ne y^{\prime} \text{ and } \text{ genus }(y) = \text{ genus }(y^{\prime}), \\ 1.25, & \text{ if } \text{ genus }(y) \ne \text{ genus }(y^{\prime}) \text{ and } \text{ family }(y) = \text{ family }(y^{\prime}), \\ 1.5, & \text{ otherwise}, \end{array} \right.$$

where $\text{ genus }(y)$ and $\text{ family }(y)$ refer to the genus and the family of *y*, respectively.

The random forest classifiers, described earlier, are originally designed to optimize accuracy. However, several methods have been described in literature where cost-sensitive classifiers are allowed to learn with asymmetric costs [32–34]. Moreover, as the cost function that we use is derived from a tree-like hierarchy on the labels, existing hierarchical classification methods [35] can be used to solve our problem as well. The methods that have been proposed in literature range from simple extensions of traditional learning algorithms, for example relying on over-sampling or threshold moving [36], to more complex dedicated hierarchical classification algorithms [37]. In this paper, we use an approach that is called a threshold moving algorithm presented by Zhou and Liu [36], and essentially is a post-processing of the predicted probability mass function over the classes, to obtain the prediction that minimizes the posterior predictive loss in a Bayesian framework [38].

As a starting point, we refer to $p(y \mid {\mathbf {x}})$ as the posterior probability that the label, i.e. the species name, of a test instance with a feature vector ${\mathbf {x}}$ is equal to *y*. We now select the label $y^*$ that minimizes the expected value of *C* under the posterior probability mass functions $p(y \mid {\mathbf {x}})$:

2 $$y^* = \arg \min _{y^{\prime} \in Y} \sum _{y \in Y} C(y, y^{\prime})\, p(y \mid {\mathbf {x}}),$$

where *Y* is the label set. During the test phase, $p(y \mid {\mathbf {x}})$ is not known but is replaced with its estimator, obtained using the random forest classifier. This approach does not require any modification of the random forest learner, as it only relies on a post-processing of the estimated probabilities from a fitted random forest model. When using the random forest classifier in the traditional way, the class with the highest estimated probability is the predicted label. Note that this estimator can still be obtained using the latter strategy by modifying *C* such that $C(y, y^{\prime})= 1$ for any $y \ne y^{\prime}$.

## Results

### Single-view versus multi-view classification

#### Performance of single-view classifiers

In this section, we discuss the advantages of multi-view classification approaches, a first batch of experiments was performed using single-view classifiers and several data augmentation techniques, resulting in the following datasets: 1000 × 1000 pixels (original), 500 × 1000 pixels (partitioned by dividing in half), 500 × 500 pixels (partitioned by dividing into four parts) and 500 × 500−OGRN (partitioned into four parts: first piece, original—O; second, smoothed—G; third, rotated—R and the last one, noisy—N). See “Data augmentation” section for more details on data augmentation.

From Table 2, it can be inferred that the transverse view is most informative for identifying the species, which agrees with other research [39]. Moreover, data augmentation helps to improve the performance. It is clear that partitioning the original image into four parts leads to an increase of the predictive performance from 0.56 to 0.75 where the size of the dataset is quadrupled replacing each 1000 × 1000 pixels image by four 500 × 500 pixels images.

**Table 2:** Accuracies obtained using single-view classifiers

Data augmentation technique	Accuracy (± std)		
	Transverse	Tangential	Radial
500 × 500	0.75 (± 0.02)	0.69 (± 0.01)	0.54 (± 0.01)
500 × 500−OGRN	0.38 (± 0.02)	0.34 (± 0.01)	0.27 (± 0.01)
500 × 1000	0.71 (± 0.02)	0.71 (± 0.01)	0.52 (± 0.01)
1000 × 1000	0.56 (± 0.02)	0.42 (± 0.02)	0.42 (± 0.02)

#### Performance of multi-view classifiers

In a second batch of experiments, the added value of using a multi-view model was investigated. Table 3 shows the results of the MVRF model (Multi-View Random Forest model) in terms of accuracy computed using 4-fold cross-validation. From these results, it is clear that the addition of LPQ features from additional anatomical planes leads to an improvement of the classification accuracy. This result shows that both the additional information that is available in the different cross-sections and type of model both contribute significantly to the performance. The best performance (0.95) is obtained using the MVRF model.

**Table 3:** Comparison of the results using the sections separately and the random forest model

	Accuracy (± std)			
	TS	TS + TLS	TS + TLS + RLS	MVRF
500 × 500	0.75 (± 0.02)	0.86 (± 0.02)	0.89 (± 0.02)	**0.95 (± 0.01)**
500 × 500−OGRN	0.38 (± 0.02)	0.48 (± 0.02)	0.51 (± 0.02)	0.62 (± 0.03)
500 × 1000	0.71 (± 0.02)	0.85 (± 0.02)	0.87 (± 0.02)	0.91 (± 0.02)
1000 × 1000	0.56 (± 0.02)	0.62 (± 0.04)	0.66 (± 0.03)	0.66 (± 0.02)

The first three columns respectively show the accuracy obtained using a random forest model trained on the LPQ features of the transverse images only (TS), a random forest model that uses the concatenation of LPQ features of the transverse and tangential sections (TS + TLS) and a random forest model that is obtained using the LPQ features from all three sections (TS + TLS + RLS)

**Fig. 5:**
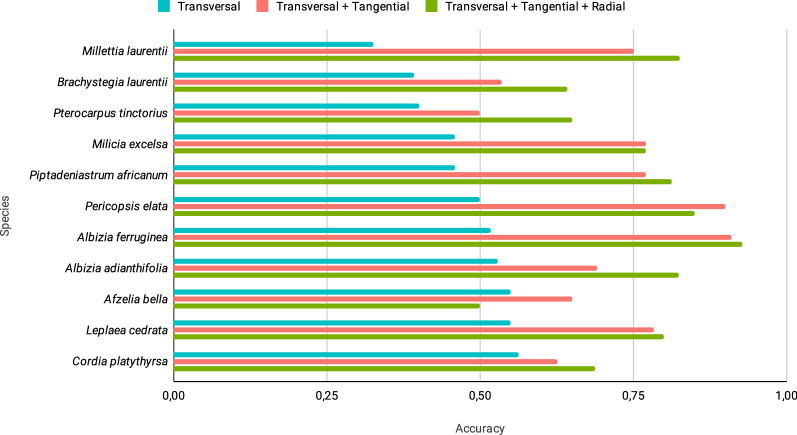
Influence of using only features from the transverse section and adding features from the tangential and radial sections

In Fig. 5, the influence of extending the features derived from the transverse section with those extracted from the tangential and radial sections is visualized per species. It can be seen here that for the eleven species that exhibited the lowest accuracy, complementing the LPQ features of the transverse section with features from the tangential and radial sections improves the classification results significantly for all species (with the exception of a small decrease for *Afzelia bella*).

#### Gaining insight into the modes of failure

**Fig. 6:**
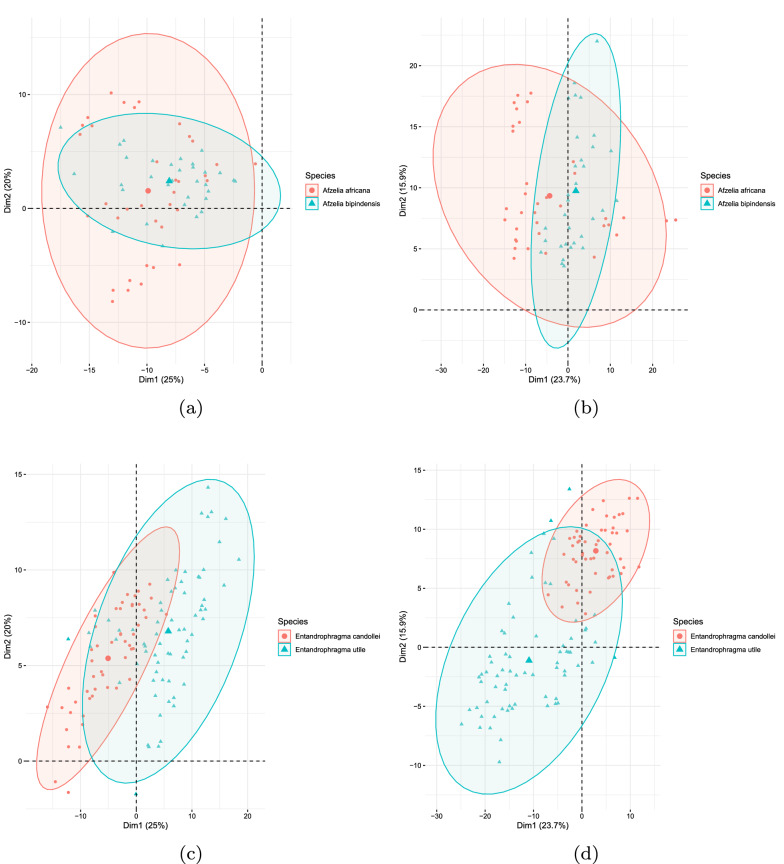
2D PCA-plot. Species *Afzelia africana* and *Afzelia bipindensis* using only features of the transverse section (**a**) and adding features of the tangential section (**b**). Species *Entandrophragma candollei* and *Entandrophragma utile* using only features of the transverse section (**c**) and adding features of the tangential section (**d**)

The results show that the overall accuracy of the classification model improves when features of additional sections are added. Hereafter, we disentangle the reasons for this. Figure 6a–d show score plots obtained after performing a principal component analysis (PCA) on the data matrix of the LPQ features (data of all 77 species). Figure 6a, c show the score plot in the PCA space when only using transverse features and Fig. 6b, d show the score plot in the PCA space computed using the concatenated feature space transverse plus tangential. For the *Afzelia africana* and *Afzelia bipindensis* shown in Fig. 6a, b, cannot easily be separated in the first two dimensions of the principal component space. This is as expected as both species cannot easily be distinguished by considering one or both sections. On the other hand, Fig. 6c, d show the score plots of *Entandrophragma candollei* and *Entandrophragma utile*. From these figures, it is clear that a better separation is observed when the LPQ features of the tangential section are added. One of the main determinants to differentiate between the two Entandrophragma species is seen only on the tangential plane. This explains that, when adding the features from the tangential section, there is a better separation of those two species. This is not the case for the *Afzelia* species, for which the tangential plane does not aid in the visual identification of these two species.

**Fig. 7:**
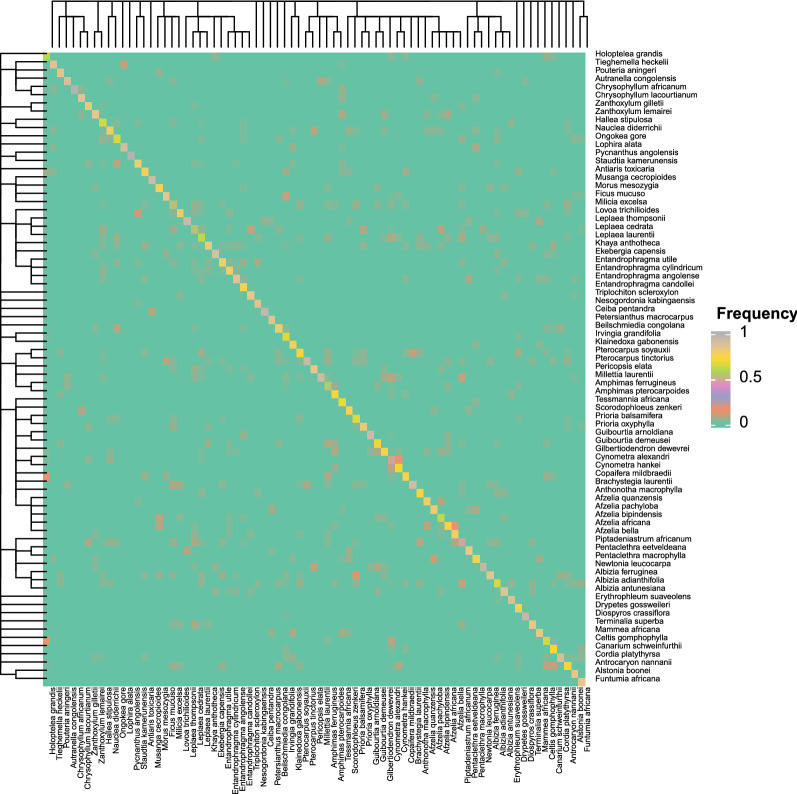
Confusion matrix for the 500 × 500 dataset using features of the transverse section

**Fig. 8:**
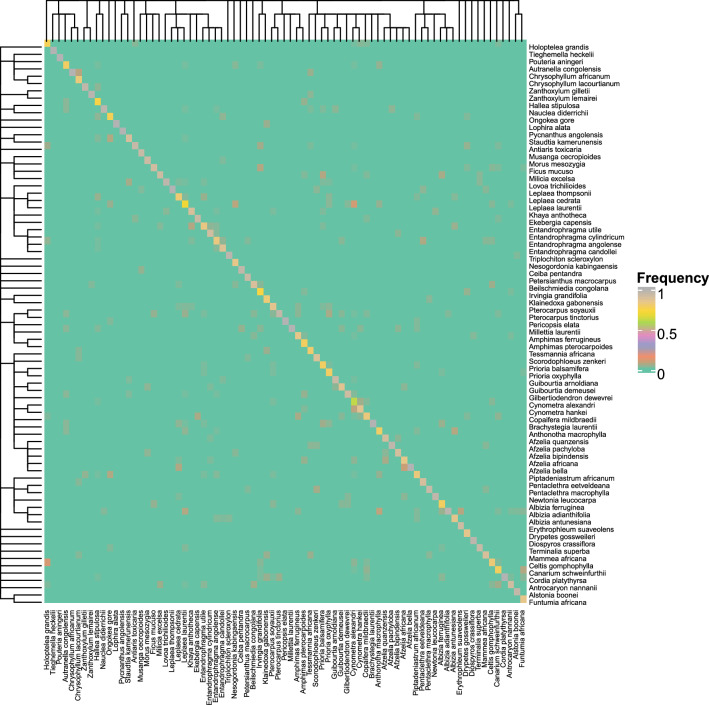
Confusion matrix for the 500 × 500 dataset using features of the transverse plus tangential section

A more complete (and more quantitative) view on the improved separability due to the addition of information on the tangential section is shown in Figs. 7 and 8. Figure 7 shows the confusion matrix for the classification of all samples using only features of the transverse section, while Fig. 8 shows the confusion matrix for the classification using features of the transverse plus tangential sections. Moreover, the phylogenetic tree based on Table 1 is added to the left and top margins. It is clear that the highest values can be found at the diagonal and no other clear patterns can be discerned. From a phylogenetic point of view, no clear overall patterns can be observed in the confusion matrix. However, this confusion matrix illustrates that, for instance, within *Afzelia*, there is quite some intra-genus confusion. A similar observation can be made for *Cynometra*. The latter confusion matrix (Fig. 8) is much cleaner, showing that the number of misclassifications decreases when adding features from the tangential section. However, there is some confusion within *Cynometra* as well.

### Including genus and family information in the classification process

In a third batch of experiments, we investigated whether including information on the phylogeny into the learning process can improve the accuracy. Table 4 shows the results. The first column (RF) shows the accuracy obtained using the random forest classifier (1000 × 1000 pixels, so without data augmentation) trained using only the features derived from the transverse section, using the species as a target. Moreover, this table also shows the accuracy of this same model at the genus and family level. The difference between these accuracies is small, implying that most of the classification errors already exist at the family level. Moreover, these results show that given a correct family identification, the probability that the species will be correctly identified is 0.63. The last row shows the average hierarchical loss $\text{ H-Loss } = \frac{1}{n}\sum _{(y, {\hat{y}})} C(y, {\hat{y}})$ (which is minimized by the cost-sensitive algorithm), where the sum runs over all couples of observed labels *y* and predicted labels ${\hat{y}}$ and *n* is the number of test cases. This loss can be seen as a hierarchical combination of the losses observed at the species, genus and family levels (the range of this average loss is [0; 1.5]).

**Table 4:** Comparison of the accuracy of the random forest classifier (RF) with the cost-sensitive random forest classifier at different hierarchical levels using the transverse section of the original dataset

	RF	Cost-sensitive RF
Accuracy at species level	**0.56**	0.52
Accuracy at genus level	**0.58**	0.56
Accuracy at family level	**0.64**	0.63
H-Loss *(lower is better)*	**0.635**	0.683

The best accuracies are bold values.

The second column shows the accuracies obtained using the cost-sensitive classification algorithm. From Table 4, it can be seen that the traditional random forest classifier consistently outperforms the cost-sensitive classifier. Even when using the H-Loss, the traditional random forest classifier outperforms its cost-sensitive version. From these results, we can conclude that this attempt to exploit the class hierarchy has a negative effect on the performance. Nevertheless, this negative result provides some insight into the wood species identification problem. Most importantly, it shows that the posterior distribution, which is estimated by the random forest classifier, is not very informative, or is very poorly estimated. Even though the mode of the distribution is quite informative (as the accuracy of the traditional classifier at the species level is rather high), the estimates of probabilities for the remaining classes are not very useful and seem hard to exploit to gain predictive power. An explanation for this negative result, as well as a step towards a solution, can be found in recent literature on distribution free uncertain quantification or conformal prediction [40]. There, it is stated that there are no guarantees that the voting mechanism of the RF classifier leads to valid estimates of the class probabilities (in a frequentist sense). Conformal prediction approaches can be used to calibrate these probability estimates to produce confidence sets guaranteed to contain the ground truth with a user-specified probability. Even though these approaches are compatible with our approach, they require an additional (hold-out) dataset that is used in the calibration step. Unfortunately, the limited size of our dataset impedes the application of these approaches.

### Experiments using the leave-*k*-trees-out approach

In this last batch of experiments, for each species, all samples (images) from the same tree were separated for the test set, making the training set completely independent from the test set. In total, 165 samples from the original dataset were used for testing and 640 samples for training. When comparing the results of this leave-*k*-trees-out approach shown in Table 5 with the accuracy obtained using the traditional cross-validation schemes, we observe a dramatic decrease. This table clearly shows that the within-tree variability is much smaller than the between-trees variability. It should be noted, however, that the number of observations per species was limited and therefore, reducing the test dataset to 165 samples will have an influence on the accuracy as well. Nevertheless, it remains striking that the performance deteriorates that strongly, which stresses the importance of performing this kind of cross-validation.

**Table 5:** Comparison of the accuracy of the leave-*k*-trees-out approach, where the test set is composed of images of trees that are not in the training set

	Accuracy (± std)	
	TS + TLS + RLS	MVRF
500 × 500	0.27 (± 0.01)	0.23 (± 0.01)
500 × 500−OGRN	0.22 (± 0.01)	0.22 (± 0.01)
500 × 1000	0.28 (± 0.01)	0.25 (± 0.01)
1000 × 1000	0.30 (± 0.01)	0.28 (± 0.01)

The experiments were performed using the concatenation of the features of the three sections (TS + TLS + RLS) and the MVRF model

In our case, as the pieces of wood were obtained at different times and regions, there is large variability across the samples. Moreover, the small number of samples per species is an important reason for the low accuracy. Additional file 1: Figure S2 shows the selected samples for training and testing for the species *Lophira alata*, where we can see considerable variability between anatomical slices from the same species. This context reinforces the need for a representative dataset, with the availability of many samples and data augmentation operations.

## Discussion

Identification at the genus and family level is important because there are many similarities between species belonging to the same genus, which may, in some cases, explain misidentification. When using the multi-view random forest model, of the 14 errors in the samples of the genus *Afzelia*, five were predicted within the same genus. When considering the 10 misidentifications of samples of the genus *Cynometra*, six samples were identified as being of another species within the same genus. Considering the *Entandrophragma* genus, six erroneously identified samples were within the same genus. Of the four misidentifications of *Afzelia bella*, three were inside the same genus and from the three misidentifications of *Afzelia bipindensis*, all were in the same family and two were in the same genus.

Following this perspective, when examining the family level, the Fabaceae–Detarioideae family shows 49 misidentifications of samples at the species level; however, 14 of them remain in the family. The Meliaceae family shows 31 misidentifications of samples at the species level, with 17 misidentifications inside the family.

Exploring the Meliaceae family, out of the 10 species analyzed, three of them achieved an accuracy of one hundred percent: *Ekebergia capensis*, *Leplaea thompsonii* and *Lovoa trichilioides*. The average accuracy, considering the 10 analyzed species of the Meliaceae family, was 95% (species level). Within *Entandrophragma* and *Khaya* there are several misidentifications. *Entandrophragma angolense*, *Entandrophragma candollei* and *Entandrophragma utile* are missclassified several times as *Khaya*. Two out of four misclassified samples from *Khaya anthotheca* were misclassified as *Entandrophragma*. Three out of six misclassified samples of *Leplaea cedrata* are misclassified as *Entandrophragma utile* and two out of four samples of *Entandrophragma utile* are misclassified as *Khaya anthotheca*.

Deklerck et al. [6] used metabolome profiles collected using Direct Analysis in Real Time (DART™) ionization coupled with Time-of-Flight Mass Spectrometry (DART-TOFMS) to analyze 95 specimens of Meliaceae. They were able to identify 82.2% of the samples using a random forest model. *Entandrophragma cylindricum* and *Entandrophragma utile* have different chemical fingerprints and could be separated. *Entandrophragma candollei* and *Entandrophragma angolense* could not be accurately differentiated and *Khaya anthotheca* was sometimes misidentified as one of these two species. This shows that, in some cases, a combination of wood anatomical analysis and DART-TOFMS will be necessary to identify a species. In addition, different techniques have different advantages. For example, chemical treatment or addition of glues to timber products might make DART-TOFMS analysis more challenging, whereas identification through wood anatomical analysis will remain unaffected.

In the work of Muellner et al. [41], six species of the Meliaceae family were identified using DNA barcoding reaching an accuracy of 67%. In Ravindran et al. [20], 10 species of the Meliaceae family were identified based on deep convolutional neural networks, achieving an accuracy of 87% at species the level and an accuracy of 96% at the genus level. Kitin et al. [42] used DART-TOFMS to study two species of *Afzelia*, *Afzelia pachyloba* and *Afzelia bipindensis*. Although the two species are not easily separated using the IAWA standard microscopic wood features, the results using DART-TOFMS reached an accuracy of 78%.

Although there are different identification methods with acceptable accuracies, so far there is no method that is fully effective for identifying all wood species. Thus, the way forward is to use a combination of different methods, such as DART-TOFMS, texture analysis and machine learning.

## Conclusions

The images obtained to perform the experiments were extracted from wood samples collected at different time periods, which may help explain differences in texture features. Weather conditions may affect the features of functional wood anatomy, such as vessel frequency and the development of the water transport pathways, making the pattern recognition task more complex.

The difficulty of obtaining wood samples is an important issue. In this way, being able to use different sections from the same sample enriches the representativeness of each sample, improving the accuracy of the classification. However, just concatenating the features of the sections is not enough, as shown in the experiments. The need arises to create a model that combines the features extracted from the three planes of section. This way, this paper presented a random forest model that uses the out of bag probabilities provided by three types of texture images, being obtained from transverse, tangential and radial section imagery. This approach showed better results than using a random forest model alone, even if the three sections are used in a concatenated way. The experiments showed that the results improved substantially when using the proposed model.

## Supplementary Information

**Additional file 1. Figure S1**: 2D PCA-plot of the class Afzelia africana for the original dataset, the dataset of original images divided in two parts, the dataset of original images divided in four parts and the dataset of original image divided in four parts with noise and rotation.
**Figure S2**: Samples of Lophira alata species. The first and second columns show samples of the training set and the third and fourth columns show samples of the test set for this species. (a)–(d) are transverse, (e)–(h) are tangential and (i)–(l) are radial sections.

## Data Availability

The dataset generated and analysed during the current study is available at 10.5281/zenodo.6611733.

## Abbreviations

CNN: Convolutional neural network
DART-TOFMS: Direct Analysis in Real Time (DARTTM) ionization coupled with Time-of-Flight Mass Spectrometry
EUTR: European Union Timber Regulation 2013
LBP: Local Binary Pattern
LPQ: Local Phase Quantization
MVRF: Multi-view random forest model
OGRN: Original (O), smoothed with Gaussian (G), rotated (R) and noisy (N)
PCA: Principal component analysis
RF: Random Forest classier
RLS: Radial longitudinal section
SVM: Support Vector Machine
TLS: Tangential longitudinal section
TS: Transverse section
