# Supplementary material for: Improved wood species identification based on multi-view imagery of the three anatomical planes
Source: Plant Methods. 2022 Jun 11;18:79. doi: 10.1186/s13007-022-00910-1 (PMC9188236; doi:10.1186/s13007-022-00910-1)
Supplement: Supplementary file 1 — Additional file 1. Figure S1: 2D PCA-plot of the class Afzelia africana for the original dataset, the dataset of original images divided in two parts, the dataset of original images divided in four parts and the dataset of original image divided in four parts with noise and rotation. Figure S2: Samples of Lophira alata species. The first and second columns show samples of the training set and the third and fourth columns show samples of the test set for this species. (a)–(d) are transverse, (e)–(h) are tangential and (i)–(l) are radial sections. [file 13007_2022_910_MOESM1_ESM.pdf]

Additional file 1

2D PCA-plot

Figure S1 shows the 2D PCA-plot of the class *Afzelia africana*. The characteristics of each image continue to be representative and distinct even though the number of samples increases. When the images are divided in two or four parts, the characteristics remain concentrated in the same region. However, when applying the smoothing, noise and rotation operations, the samples tend to spread out, making it difficult to identify them. However, it is important to carry out these operations on the data because they can represent real situations, since the wood samples can be obtained at different times or in adverse situations, and different regions.

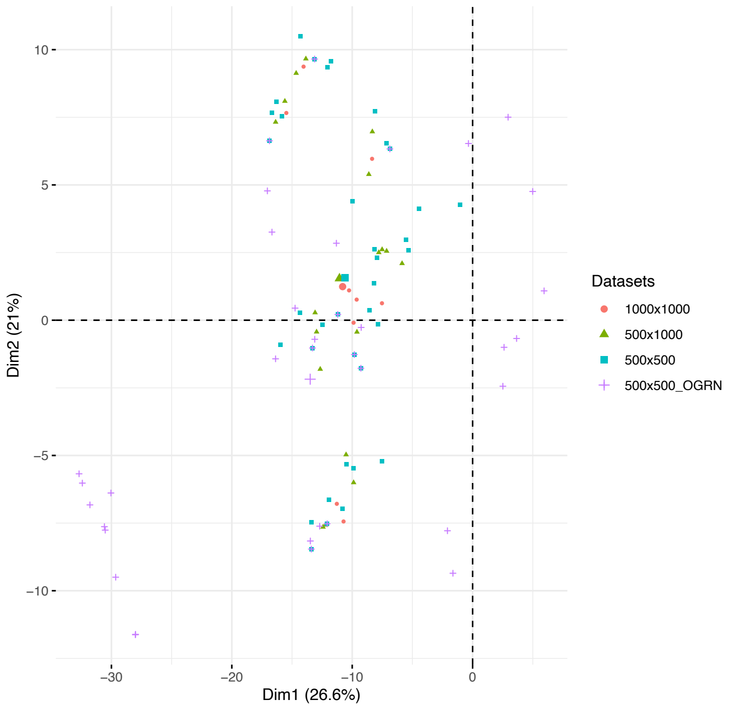

Fig. S1: 2D PCA-plot of the class *Afzelia africana* for the original dataset, the dataset of original images divided in two parts, the dataset of original images divided in four parts and the dataset of original image divided in four parts with noise and rotation.

Variability of training and testing samples

Figure S2 shows samples of *Lophira alata* species to emphasize the variability between samples from different wood samples.

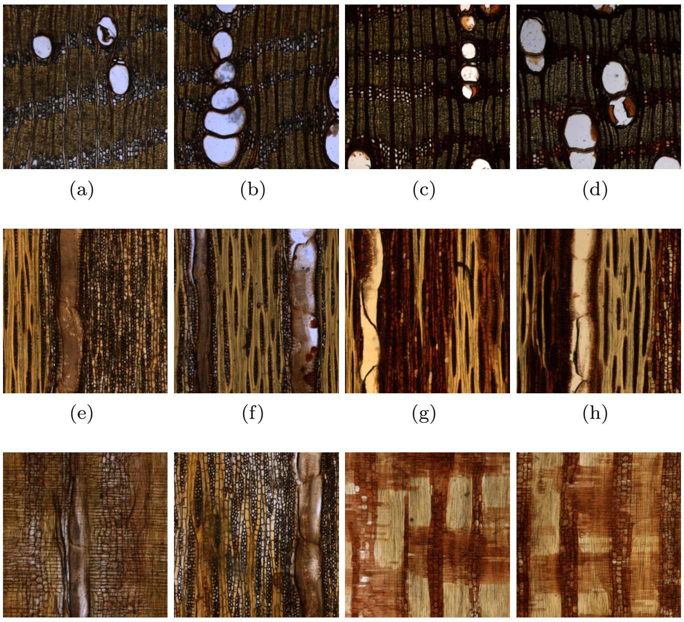

(a) (b) (c) (d) (e) (f) (g) (h) (i) (j) (k) (l)

Fig. S2: Samples of *Lophira alata* species. The first and second columns show samples of the training set and the third and fourth columns show samples of the test set for this species. (a)–(d) are transverse, (e)–(h) are tangential and (i)–(l) are radial sections.
